# Lower Cardiac Output Relates to Longitudinal Cognitive Decline in Aging Adults

**DOI:** 10.3389/fpsyg.2020.569355

**Published:** 2020-11-09

**Authors:** Corey W. Bown, Rachel Do, Omair A. Khan, Dandan Liu, Francis E. Cambronero, Elizabeth E. Moore, Katie E. Osborn, Deepak K. Gupta, Kimberly R. Pechman, Lisa A. Mendes, Timothy J. Hohman, Katherine A. Gifford, Angela L. Jefferson

**Affiliations:** ^1^Vanderbilt Memory & Alzheimer’s Center, Department of Neurology, Vanderbilt University Medical Center, Nashville, TN, United States; ^2^Vanderbilt Brain Institute, Vanderbilt University, Nashville, TN, United States; ^3^Vanderbilt University School of Medicine, Vanderbilt University, Nashville, TN, United states; ^4^Department of Biostatistics, Vanderbilt University Medical Center, Nashville, TN, United States; ^5^Division of Cardiovascular Medicine, Department of Medicine, Vanderbilt University Medical Center, Nashville, TN, United States; ^6^Vanderbilt Heart Imaging Core Lab, Vanderbilt Translational and Clinical Cardiovascular Research Center, Vanderbilt University Medical Center, Nashville, TN, United States; ^7^Department of Neurology, Vanderbilt University Medical Center, Nashville, TN, United States; ^8^Vanderbilt Genetics Institute, Vanderbilt University Medical Center, Nashville, TN, United States

**Keywords:** apolipoprotein E ε4, cognitive decline, aging, mild cognitive impairment, cardiac function

## Abstract

**Background:**

Subclinical reductions in cardiac output correspond to lower cerebral blood flow (CBF), placing the brain at risk for functional changes.

**Objectives:**

This study aims to establish the consequences of reduced cardiac output on longitudinal cognitive outcomes in aging adults.

**Methods:**

Vanderbilt Memory and Aging Project participants free of clinical dementia and heart failure (*n* = 306, 73 ± 7, 58% male) underwent baseline echocardiography to assess cardiac output (L/min) and longitudinal neuropsychological assessment at baseline, 18 months, 3 and 5 years. Linear mixed-effects regressions related cardiac output to trajectory for each longitudinal neuropsychological outcome, adjusting for age, sex, race/ethnicity, education, body surface area, Framingham Stroke Risk Profile score, apolipoprotein E (*APOE*) ε4 status and follow-up time. Models were repeated, testing interactions with cognitive diagnosis and *APOE-*ε4 status.

**Results:**

Lower baseline cardiac output related to faster declines in language (β = 0.11, *p* = 0.01), information processing speed (β = 0.31, *p* = 0.006), visuospatial skills (β = 0.09, *p* = 0.03), and episodic memory (β = 0.02, *p* = 0.001). No *cardiac output x cognitive diagnosis* interactions were observed (*p* > 0.26). *APOE*-ε4 status modified the association between cardiac output and longitudinal episodic memory (β = 0.03, *p* = 0.047) and information processing speed outcomes (β = 0.55, *p* = 0.02) with associations stronger in *APOE-*ε4 carriers.

**Conclusion:**

The present study provides evidence that even subtle reductions in cardiac output may be associated with more adverse longitudinal cognitive health, including worse language, information processing speed, visuospatial skills, and episodic memory performances. Preservation of healthy cardiac functioning is important for maintaining optimal brain aging among older adults.

## Introduction

Altered cardiac hemodynamics have been associated with cerebral blood flow (CBF) disturbances (Jefferson et al., 2017), smaller brain volumes (Sabayan et al., 2015), and poorer cognitive performances (Sabayan et al., 2015; Kresge et al., 2018), particularly among *APOE*-ε4 carriers (Bown et al., 2019). Prior studies, however, have been cross-sectional (Sabayan et al., 2015; Jefferson et al., 2017; Kresge et al., 2018; Bown et al., 2019) with limited investigation into the connection between subclinical cardiac dysfunction and longitudinal consequences, especially cognitive trajectories among aging adults. Given cross-sectional evidence, associations between cardiac function and longitudinal cognition may exist.

Reduced cardiac output is associated with reduced CBF (Jefferson et al., 2017) which may result in damaging cascades in the brain due to inadequate nutrient delivery and oxidative stress. The medial temporal lobes (Chen et al., 2011; Roy et al., 2017) as well as the superior frontal and orbito-frontal gyri (Chen et al., 2011; Roy et al., 2017) are susceptible to reductions in blood flow with aging (Chen et al., 2011) and heart failure (Roy et al., 2017), and the hippocampus, a structure of the medial temporal lobes, is vulnerable to blood brain barrier breakdown with aging (Montagne et al., 2015). Of course, in addition to regional susceptibility to reduced blood supply, certain regions demand more energy which creates additional vulnerabilities (Hawkins et al., 1979; Payabvash et al., 2011). Cognitive functions specific to regions of the brain that are particularly susceptible to blood flow reductions may be compromised in the context of hemodynamic dysregulation. The medial temporal lobes and frontal-subcortical networks, which are regions sensitive to hypoperfusion, are responsible for episodic memory (Tulving and Markowitsch, 1998), executive function (Cummings, 1998), and information processing (Cummings, 1998) and therefore these functions are vulnerable to subtle but chronic hemodynamic dysregulation over time.

This study aimed to characterize the association between subclinical cardiac dysfunction and cognitive trajectory in aging adults. Specifically, we examined whether baseline cardiac output relates to longitudinal cognitive performance among older adults free of clinical dementia, stroke, or heart failure at study entry. Based on prior work (Jefferson et al., 2010; Sabayan et al., 2015; Kresge et al., 2018), we hypothesized lower baseline cardiac output would relate to worse cognitive trajectories over the follow-up period, especially episodic memory as assessed by the Biber Figure Learning Test (Glosser et al., 2002) and California Verbal Learning Test-II (Delis et al., 2000), information processing speed as assessed by the Delis Kaplan Executive Function System (DKEFS) Number Sequencing Test (Delis et al., 2001) and Wechsler Adult Intelligence Scale 4th Edition (WAIS-IV) Digit Symbol Test (Wechsler, 2008), and executive function as assessed by the DKEFS Number-letter Switching (Delis et al., 2001), DKEFS Tower Test (Delis et al., 2001), DKEFS Color-Word Inhibition (Delis et al., 2001), and Letter Fluency Test (Benton et al., 1994). We investigated whether associations would be modified by the *apolipoprotein E* (*APOE*) ε4 allele or cognitive diagnosis. While *APOE*-ε4 is a genetic susceptibility marker for AD (Martins et al., 2005) it is also a molecular moderator of vascular damage (Halliday et al., 2016). Thus, we hypothesized that *APOE*-ε4 carriers would have stronger associations between baseline cardiac output and cognitive decline compared to non-carriers. We also hypothesized that participants with mild cognitive impairment (MCI), a clinical prodrome of dementia, would have stronger associations compared to participants with normal cognition due to extensive neuropathology underlying cognitive symptoms. The presumed neuropathology of MCI participants disrupts normal physiology and creates vulnerability to hemodynamic dysfunction.

## Materials and Methods

### Study Cohort

The Vanderbilt Memory and Aging Project (VMAP) is a longitudinal study investigating vascular health and aging. Inclusion required participants to be age 60 years or older, speak English, have intact auditory and visual acuity, and have a study partner. Exclusion criteria included MRI contraindication, history of neurological disease (e.g., stroke, epilepsy), prevalent heart failure, major psychiatric illness, head injury with loss of consciousness greater than 5 min and systemic or terminal illness (e.g., cancer) that could adversely affect follow-up participation.

**FIGURE 1:**
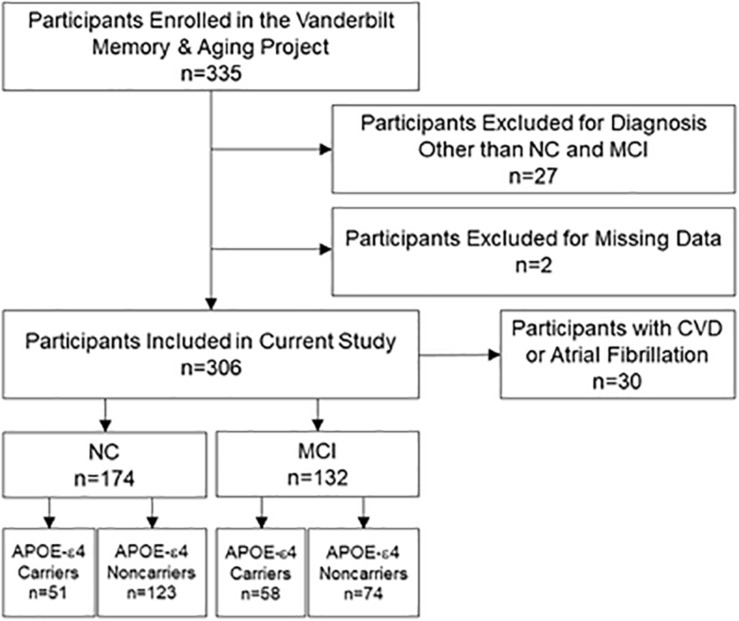
Participant inclusion and exclusion details. Missing data categories at baseline are mutually exclusive and included missing at least one neuropsychological outcome (*n* = 1) and missing covariate data (*n* = 1). Sensitivity analyses excluded participants with CVD or atrial fibrillation. *APOE*-ε4, apolipoprotein E ε4. CVD, cardiovascular disease. MCI, mild cognitive impairment. NC, normal cognition.

At eligibility, participants completed a medical history and record review, Clinical Dementia Rating interview, and neuropsychological assessment. Participants were enrolled if they had normal cognition (NC) or met diagnostic criteria for MCI which requires all of the following criteria be fulfilled: (a) a cognitive concern by a participant, informant, or clinician, (b) impairment in at least one cognitive domain, (c) independence in functional abilities, and (d) no dementia (Albert et al., 2011). At enrollment, each participant completed a comprehensive evaluation, including fasting blood draw, physical examination, clinical interview, medication review, neuropsychological assessment, echocardiogram, and cardiac magnetic resonance. Identical procedures were repeated at each time point, including 18 months, 3 and 5 years, for longitudinal follow-up of the cohort. Participants were excluded from the current study for missing baseline echocardiogram, baseline covariate data, or neuropsychological data across all timepoints. See Figure 1 for participant selection details. Three hundred and six participants included in the study were seen at baseline and 278 had more than one timepoint of data for a mean follow-up period of 3.5 ± 1.5 years. Two hundred and seventy participants were seen at 18-months, 256 participants at 3-year, and 133 participants at 5-year follow-ups.

### Standard Protocol Approvals, Registrations, and Participant Consents

The protocol was approved by the Vanderbilt University Medical Center Institutional Review Board. Written informed consent was obtained from all participants at each time point prior to data collection. Due to participant consent restrictions in data sharing, a subset of data is available for purposes of reproducing results or replicating procedures. Data, analytic methods, and study materials can be obtained by contacting the corresponding author.

### Echocardiography

Standard 2-dimensional, M-mode, and Doppler transthoracic echocardiography was performed by a single research sonographer at the Vanderbilt University Medical Center Clinical Research Center on a Philips IE33 cardiac ultrasound machine (Philips Medical, Andover, MD). Digital images with measurements were confirmed by board-certified cardiologists (DKG, LAM) using commercially available software (HeartLab; AGFA Healthcare, Greenville, SC). All raters were blinded to clinical information.

Image acquisition and quantification were performed according to standards from the American Society of Echocardiography (Lang et al., 2015). Stroke volume was calculated from the left ventricular outflow tract velocity time integral and diameter, and cardiac output was calculated as stroke volume x heart rate. Final values were derived from a single cardiac cycle for participants in normal sinus rhythm or the average of three consecutive cardiac cycles in the presence of atrial fibrillation.

### Neuropsychological Assessment

All participants underwent a comprehensive neuropsychological protocol at each time point, including Boston Naming Test (Kaplan et al., 1983), Animal Naming (Goodglass and Kaplan, 1983), WAIS-IV Coding (Wechsler, 2008), DKEFS Number Sequencing (Delis et al., 2001), DKEFS Tower Test (Delis et al., 2001), DKEFS Color-Word Inhibition (Delis et al., 2001), DKEFS Number-Letter Switching (Delis et al., 2001), Letter Fluency (FAS) (Benton et al., 1994), Hooper Visual Organization Test (Hooper, 1983), Biber Figure Learning Test (Glosser et al., 2002), and California Verbal Learning Test-II (Delis et al., 2000). Measures were carefully selected to preclude floor or ceiling effects and were not utilized for screening, diagnosis, or selection of participants for the study. To minimize multiple comparisons, composite z-scores were derived separately for episodic memory and executive function performances which leveraged item-level data from the multiple tests mentioned above to create bifactor latent variable models which fit the data well (i.e., root mean square error of approximation: 0.09 for memory and 0.03 for executive function) (Kresge et al., 2018).

### Analytical Plan

Body surface area was calculated according to the DuBois formula (Du Bois and Du Bois, 1989): weight (kg)^0.425^ × height (cm)^0.725^ × 0.007184. Systolic blood pressure was the mean of two measurements. Diabetes mellitus included fasting blood glucose ≥ 126 mg/dL, hemoglobin A_1c_ ≥ 6.5%, or oral hypoglycemic or insulin medication use. Medication review determined anti-hypertensive medication usage. Left ventricular hypertrophy was determined using echocardiogram as left ventricle mass index > 95 g/m^2^ in women or > 115 g/m^2^ in men. Atrial fibrillation included self-report with supporting evidence from echocardiogram, cardiac magnetic resonance, or prior surgery or medication usage to treat atrial fibrillation. Self-report was used to ascertain current cigarette smoking (yes/no within the year before baseline). Prevalent CVD from self-report with supporting medical record evidence included angina, coronary heart disease, or myocardial infarction (heart failure was an exclusion criterion at study enrollment). Framingham Stroke Risk Profile (FSRP) score was calculated by applying points by sex for age, systolic blood pressure accounting for anti-hypertensive medication usage, diabetes mellitus, current cigarette smoking, left ventricular hypertrophy, prevalent CVD, and atrial fibrillation (D’Agostino et al., 1994). *APOE* genotyping was performed using a TaqMan assay on DNA extracted from whole-blood samples as described previously (Jefferson et al., 2016), and *APOE*-ε4 carrier status was defined as positive (ε2/ε4, ε3/ε4, ε4/ε4) or negative (ε2/ε2, ε2/ε3, ε3/ε3).

Linear mixed-effects regression models related baseline cardiac output to longitudinal neuropsychological performance (one test per model), including an interaction with time to follow-up between baseline and last follow-up visit (in years) as the term of interest. We model the trajectory of cognition using these linear mixed-effect regression models, where terms involving follow-up time capture cognitive decline. Based on known associations with cardiovascular function and brain health, a series of covariates were identified a priori, including age (Wisdom et al., 2012), sex (Prabhavathi et al., 2014; McCarrey et al., 2016), education (Chen et al., 2019), race/ethnicity (Leigh et al., 2016), FSRP (excluding points for age) (Wolf et al., 1991; D’Agostino et al., 1994), and *APOE*-ε4 carrier status (Rawle et al., 2018). Additional covariates included body surface area (to adjust for individual differences in cardiac output) and follow-up time. Sensitivity analyses were performed excluding participants with prevalent CVD and atrial fibrillation, adjusting for cognitive diagnosis as well as outliers above 4 standard deviations. To test hypotheses related to *APOE*-ε4 status, models were repeated with a cardiac output x follow-up time x *APOE*-ε4 carrier status interaction term with follow-up models stratified by *APOE*-ε4 carrier status (*APOE*-ε4 carrier and non-carrier) with identical covariates. To test hypotheses related to cognitive diagnosis, models were repeated with a cardiac output x follow-up time x cognitive diagnosis interaction term with follow-up models stratified by cognitive diagnosis (NC and MCI). Lower order terms were included in all interaction models. To determine if outliers were driving the results, additional models were calculated excluding predictor or outcome values > 4 standard deviations from the group mean. Significance was set *a priori* at *p* < 0.05. False discovery rate (FDR) correction for multiple comparisons was performed using the Benjamini-Hochberg procedure adjusting for seven tests evaluated in planned analyses. All analyses were conducted using R 3.5.2^1^.

## Results

### Participant Characteristics

The final sample included 306 participants with 174 NC and 132 MCI participants. The mean sample age at baseline was 73 ± 7 years (ranging 60–92 years), 58% were men, and 87% self-identified as non-Hispanic white. Cardiac output values ranged 2.0–8.7 L/min (5.0 ± 1.3) and did not differ between NC and MCI participants (*p* = 0.58). Sample characteristics are presented in Table 1. As expected, the MCI participants had worse baseline performance on all neuropsychological measures (Table 1) and greater annual longitudinal decline across all measures compared to NC participants (Table 2). Of the 306 participants included in the current study, 278 had more than one time point of data with a mean 3.5 ± 1.5 year follow-up for the sample which differed by participant group (NC = 3.8 ± 1.4, MCI = 3.0 ± 1.6, *p* < 0.001). Individuals with one time point of data (*n* = 28) were on average older (*p* = 0.004), less educated (*p* < 0.001), had a higher Framingham Stroke Risk Profile score (*p* = 0.02), and performed worse on all baseline cognitive measures (*p* < 0.03) than participants with multiple time points. Over the follow-up period, 33 participants developed incident dementia (defined as a change in Clinical Dementia Rating global score), eight experienced incident stroke, and two participants developed incident heart failure.

**TABLE 1:** Participant characteristics at study entry.

	**Total (*n* = 306)**	**NC (*n* = 174)**	**MCI (*n* = 132)**	***p*-value**
**Demographic and health characteristics**				
Age, years	73 ± 7	72 ± 7	73 ± 8	0.37
Sex, % male	58	59	56	0.58
Race, % non-Hispanic white	87	87	86	0.66
Education, years	16 ± 3	16 ± 2	15 ± 3	**<0.001**
*APOE-*ε4 carriers, %	36	29	44	**0.008**
Montreal Cognitive Assessment	25.3 ± 3.4	27.0 ± 2.2	23.1 ± 3.4	**<0.001**
Body surface area, m^2^	1.9 ± 0.2	1.9 ± 0.2	1.9 ± 0.2	0.51
Framingham Stroke Risk Profile score, total	12.4 ± 4.3	12.0 ± 4.3	13.0 ± 4.3	**0.04**
Systolic blood pressure, mmHg	142 ± 18	140 ± 17	145 ± 19	**0.02**
Anti-hypertensive medication usage, %	55	53	56	0.65
Diabetes, %	18	16	21	0.25
Smoking, % current	2	2	3	0.45
Left ventricular hypertrophy, %	5	3	6	0.28
Prevalent CVD, %	5	6	3	0.26
Atrial fibrillation, %	6	6	7	0.70
Cardiac output, L/min	5.0 ± 1.3	5.0 ± 1.3	4.9 ± 1.3	0.58
Time to follow up, years	3.5 ± 1.5	3.8 ± 1.4	3.0 ± 1.6	**<0.001**
**Neuropsychological performances**				
Boston Naming Test, 30-Item	26.8 ± 3.2	27.9 ± 2.0	25.4 ± 3.9	**<0.001**
Animal Naming	18.9 ± 5.5	21.0 ± 4.9	16.2 ± 5.2	**<0.001**
WAIS-IV Coding	53 ± 13	57 ± 12	46 ± 12	**<0.001**
DKEFS Number Sequencing*	43 ± 21	36 ± 13	51 ± 26	**<0.001**
Executive Function Composite	−0.004 ± 0.9	0.4 ± 0.6	−0.6 ± 0.9	**<0.001**
Hooper Visual Organization Test	24.4 ± 3.2	25.3 ± 2.4	23.2 ± 3.6	**<0.001**
Episodic memory composite	0.003 ± 1.0	0.6 ± 0.7	−0.7 ± 0.8	**<0.001**

Descriptive statistics by baseline diagnosis were calculated using mean ± standard deviation for continuous variables and frequencies for categorical variables. Between-group characteristics were statistically compared using Wilcoxon test for continuous variables and Pearson test for categorical variables. Bolded values represent significant findings. Framingham Stroke Risk Profile (minus age) scores are 6.5 ± 3.2 for the combined cohort (NC = 6.2 ± 3.1 and MCI = 6.9 ± 3.3). All neuropsychological performance values are total correct excluding timed tasks measured in seconds (s). *Higher values reflect worse performance. APOE-ε4, apolipoprotein E ε4 allele; CVD, cardiovascular disease; DKEFS, Delis-Kaplan Executive Function System; MCI, mild cognitive impairment; NC, normal cognition; WAIS-IV, Wechsler Adult Intelligence Scale, 4th edition.

**TABLE 2:** Annual change in neuropsychological performances.

	**Total (n = 278)**	**NC (n = 165)**	**MCI (n = 113)**	***p*-value**
Boston Naming Test, 30-Item	−0.2 ± 0.9	0.04 ± 0.4	−0.5 ± 1.3	**<0**.**001**
Animal Naming	−0.5 ± 1.3	−0.3 ± 1.3	−0.8 ± 1.4	**0**.**003**
WAIS-IV Coding	−1.2 ± 3.1	−0.4 ± 2.0	−2.4 ± 4.0	**<0**.**001**
DKEFS Number Sequencing*	2.9 ± 11	0.9 ± 3.6	5.9 ± 16.0	**<0**.**001**
Executive Function Composite	−0.06 ± 0.2	−0.02 ± 0.1	−0.1 ± 0.3	**0**.**005**
Hooper Visual Organization Test	−0.1 ± 1.0	0.02 ± 0.5	−0.4 ± 1.4	**0**.**03**
Episodic Memory Composite	−0.02 ± 0.2	0.02 ± 0.2	−0.08 ± 0.2	**<0**.**001**

Neuropsychological performance values represent the difference between last follow-up visit and baseline visit performances. p-values are presented for comparisons between NC and MCI groups. Bolded values represent significant findings. All neuropsychological performance values are total correct excluding timed tasks measured in seconds (s). *Higher values reflect worse performance. DKEFS, Delis-Kaplan Executive Function System; MCI, mild cognitive impairment; NC, normal cognition; WAIS-IV, Wechsler Adult Intelligence Scale, 4th edition.

### Cardiac Output and Longitudinal Neuropsychological Performances

**FIGURE 2:**
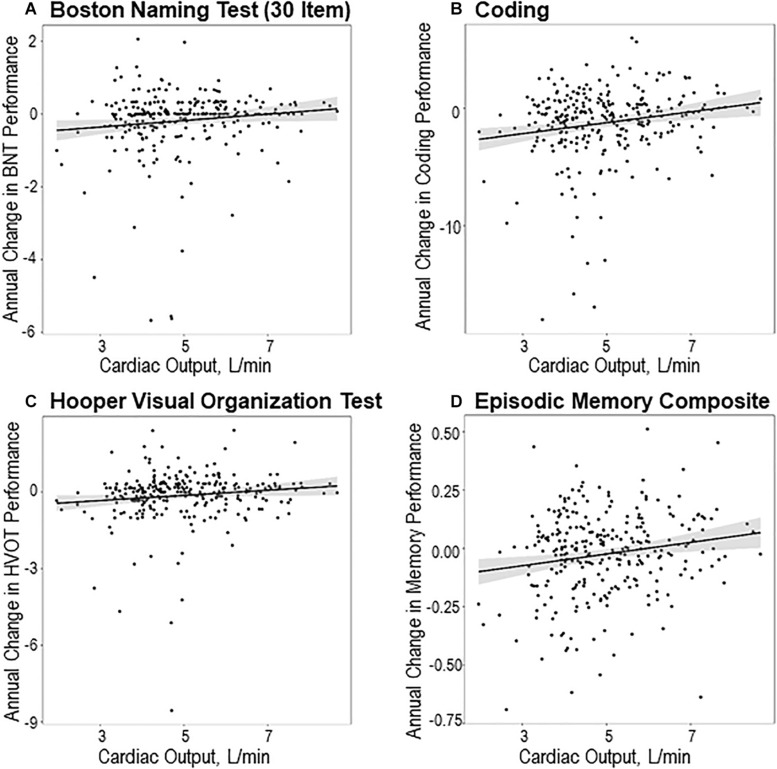
Cardiac output and longitudinal neuropsychological performances. Cardiac output and longitudinal neuropsychological performances, including **(A)** Boston Naming Test (30-Item), **(B)** Coding, **(C)** Hooper Visual Organization Test, and **(D)** episodic memory composite. Depicted are unadjusted scatter plots. Solid black line reflects fitted (predicted) values of annual change in cognitive outcomes (*y*-axis) corresponding to baseline cardiac output (*x*-axis). Shading reflects 95% confidence interval. All displayed plots are statistically significant (*p* < 0.05) and all models except for Hooper Visual Organization Test (*p* = 0.05) survive correction for false discovery rate. All participants in primary analyses are included in the scatter plots.

Lower baseline cardiac output related to faster decline over the follow-up (defined as a negative trajectory of cognition, where terms involving follow-up time capture cognitive decline) for Boston Naming Test (β = 0.11, *p* = 0.01), Coding (β = 0.31, *p* = 0.006), Hooper Visual Organization Test (β = 0.09, *p* = 0.03), and episodic memory composite performances (β = 0.02, *p* = 0.001). Cardiac output was unrelated to longitudinal trajectory for the remaining neuropsychological measures (*p* > 0.12). See Table 3 for details and Figure 2 for illustrations of significant results. In sensitivity analyses excluding participants with prevalent CVD and atrial fibrillation, as well as excluding outliers, results were similar (data not shown). Results were similar when including cognitive diagnosis as a covariate. Results continued to meet the significance threshold when correcting for FDR using the Benjamini-Hochberg procedure except for Hooper Visual Organization Test (*p* = 0.05).

**TABLE 3:** Main effect results relating baseline cardiac output to longitudinal neuropsychological performances.

	**β**	**95% Confidence interval**	***p*-value**
Boston Naming Test, 30-Item	0.11	0.02, 0.20	0.01*
Animal Naming	0.09	−0.02, 0.21	0.12
WAIS-IV Coding	0.31	0.09, 0.53	0.006*
DKEFS Number Sequencing^†^	–0.33	−0.96, 0.31	0.31
Executive Function Composite	0.008	−0.009, 0.03	0.36
Hooper Visual Organization Test	0.09	0.01, 0.18	**0.03**
Episodic Memory Composite	0.02	0.01, 0.04	0.001*

Neuropsychological performance values represent the difference between last follow-up visit and baseline visit performances. Data presented as interaction term (cardiac output x time to follow-up). Analyses performed on n = 306 participants. Models were adjusted for age, sex, race/ethnicity, education, body surface area, Framingham Stroke Risk Profile minus age, and APOE-ε4 status. Bolded values represent significant findings. All neuropsychological performance values are total correct excluding timed tasks measured in seconds (s). *Models that meet the significance threshold after applying the Benjamini-Hochberg procedure. ^†^Higher values reflect worse performance. DKEFS, Delis-Kaplan Executive Function System; WAIS-IV, Wechsler Adult Intelligence Scale, 4th edition.

### Cardiac Output × Cognitive Diagnosis and Longitudinal Neuropsychological Performances

No *cardiac output* × *cognitive diagnosis* interactions were observed for any longitudinal neuropsychological performances (*p* > 0.26). See Supplementary Table 1. Stratified models of participants with MCI were similarly null (*p* > 0.20) except for the episodic memory composite (β = 0.03, *p* = 0.02), which does not meet the significance threshold when correcting for FDR. Stratified models in NC participants revealed an association between lower baseline cardiac output and faster decline on Boston Naming Test (β = 0.07, *p* = 0.003), Coding (β = 0.26, *p* = 0.02), and Hooper Visual Organization Test performances (β = 0.06, *p* = 0.02). All of these results meet the significance threshold when correcting for FDR. Results were similar when excluding participants for prevalent CVD and atrial fibrillation, when including cognitive diagnosis as a covariate, and when excluding outliers (data now shown).

### Cardiac Output × APOE-ε4 and Longitudinal Neuropsychological Performances

*Cardiac output* × *APOE-ε4 carrier status* modified the association between cardiac output and longitudinal Coding (β = 0.55, *p* = 0.02) and episodic memory composite performances (β = 0.03, *p* = 0.047) but neither of these measures met the significance threshold when correcting for FDR. See Table 4 for details and Supplementary Figure 1 for illustrations. No interactions were observed for the remaining measures (*p* > 0.08). Among the *APOE*-ε4 positive participants, stratified results suggested lower baseline cardiac output was related to faster decline on Coding (β = 0.71, *p* = 0.01) and episodic memory composite performances (β = 0.05, *p* = 0.004). Both findings survive correction for FDR. Among the *APOE*-ε4 negative subgroup, cardiac output was unrelated to any neuropsychological performance (*p* > 0.07). Significant *APOE*-ε4 interaction models were attenuated when excluding participants with prevalent CVD and atrial fibrillation but remained when excluding outliers. Results for models stratified by *APOE*-ε4 status were similar when excluding participants for prevalent CVD and atrial fibrillation (data not shown), and when excluding outliers. When including diagnosis as a covariate an additional significant interaction with *APOE*-ε4 status was found with Boston Naming Test (β = 0.19, *p* = 0.047).

**TABLE 4:** Cardiac output ×*APOE*-ε4 carrier status interaction and stratified models.

	**β**	**95% Confidence interval**	***p*-value**
**Cardiac output** ×***APOE*-ε4 interaction**			
Boston Naming Test, 30-Item	0.17	−0.02, 0.35	0.08
Animal Naming	0.06	−0.19, 0.31	0.64
WAIS-IV Coding	0.55	0.08, 1.0	**0.02**
DKEFS Number Sequencing^†^	–0.25	−1.6, 1.1	0.72
Executive Function Composite	–0.01	−0.05, 0.03	0.58
Hooper Visual Organization Test	0.09	−0.09, 0.27	0.33
Episodic Memory Composite	0.03	0.0005, 0.06	**0.047**
**Stratified by *APOE*-ε4 Carriers**			
Boston Naming Test, 30-Item	0.21	−0.01, 0.43	0.06
Animal Naming	0.08	−0.16, 0.33	0.50
WAIS-IV Coding	0.71	0.17, 1.2	0.01*
DKEFS Number Sequencing^†^	–0.06	−1.7, 1.5	0.94
Executive Function Composite	0.002	−0.04, 0.04	0.94
Hooper Visual Organization Test	0.14	−0.05, 0.33	0.15
Episodic Memory Composite	0.05	0.02, 0.08	0.004*
**Stratified by *APOE*-ε4 Non-carriers**			
Boston Naming Test, 30-Item	0.05	−0.01, 0.11	0.12
Animal Naming	0.08	−0.04, 0.20	0.19
WAIS-IV Coding	0.12	−0.07, 0.32	0.22
DKEFS Number Sequencing^†^	–0.39	−1.0, 0.21	0.21
Executive Function Composite	0.008	−0.005, 0.02	0.24
Hooper Visual Organization Test	0.07	−0.005, 0.14	0.07
Episodic Memory Composite	0.01	−0.002, 0.03	0.10

Neuropsychological performance values represent the difference between last follow-up visit and baseline visit performances. Data presented as interaction term (cardiac output x time to follow-up). Analyses performed on 306 participants. Models were adjusted for age, sex, race/ethnicity, education, body surface area, Framingham Stroke Risk Profile minus age, and APOE-ε4 status. Bolded values represent significant findings. All neuropsychological performance values are total correct excluding timed tasks measured in seconds (s). *Models that meet the significance threshold after applying the Benjamini-Hochberg procedure. ^†^Higher values reflect worse performance. APOE-ε4, apolipoprotein E ε4 allele; DKEFS, Delis-Kaplan Executive Function System; WAIS-IV, Wechsler Adult Intelligence Scale, 4th edition.

## Discussion

Among community-dwelling older adults free of clinical dementia, stroke, and heart failure, lower cardiac output at study entry related to faster decline in language, information processing speed, visuospatial skill, and episodic memory over the mean 3.5-year follow-up period. Cognitive diagnosis did not modify these associations, but a modest 3-way interaction amongst cardiac output, follow-up time, and *APOE*-ε4 was found, such that lower cardiac output at study entry was associated with worse longitudinal trajectory for information processing speed and episodic memory performances in *APOE*-ε4 carriers.

Our observations are among the first to extend prior cross-sectional findings that lower resting cardiac output relates to worse cognitive performance in community-dwelling older adults (Jefferson et al., 2010; Sabayan et al., 2015; Bown et al., 2019) by providing evidence that subtle reductions in cardiac output also relate to faster cognitive decline in domains of language, information processing, visuospatial skill, and episodic memory. Subtle reductions in cardiac output correspond to reduced CBF delivery as recently reported by our group (Jefferson et al., 2017). Chronic lower blood flow delivery to the brain could create a gradual metabolic energy crisis for neurons (Shuang Wan et al., 2016) and oxidative stress (Cacciamani et al., 2017) that concurrently promotes increased tau phosphorylation (Melov et al., 2007), mitochondrial dysfunction (Lu et al., 2004), and astrocyte dysregulation (Furuta et al., 2017). These changes are known to drive neurodegeneration, but lower blood flow could affect the brain through other pathways by inducing blood-brain barrier dysregulation (Krueger et al., 2017) or neuroinflammation (Miyanohara and Kakae, 2018), both of which might contribute to clinical symptoms and subsequent dementia. These possible pathways align with prior epidemiological evidence linking lower levels of cardiac output to incident dementia (Jefferson et al., 2015). The present study serves to further highlight the importance of maintaining a cardiovascular healthy lifestyle and its translation to enhanced brain health.

Tests of language, information processing speed, visuospatial skills, and episodic memory were all implicated in main effect models relating baseline cardiac output to longitudinal cognitive trajectory. Language abilities generally localize to the temporal lobes (Spitsyna et al., 2006) but the Boston Naming Test, a word-retrieval task implicated in this study, requires the convergence of semantic memory (Schwartz, 2014), lexical selection (Schwartz, 2014) and visual-perceptual processing (Soble et al., 2016) and can be indicative of a global phenomenon. Still, there may be specific brain regions that are more susceptible to hemodynamic fluctuations than others. For example, regions that are likely to have higher metabolic energy demand (Hawkins et al., 1979; Payabvash et al., 2011) or are located in territories that are difficult to perfuse are more vulnerable (Howard et al., 1987). A large portion of energy expenditure in the brain occurs at synapses (Attwell and Laughlin, 2001) and regions with high neuronal density could be more vulnerable to oligemia or hypoperfusion. The temporal lobes mediate memory and language functions, have higher synaptic density in older adults with normal cognition (Brown et al., 1998), and have a less extensive network of collateral blood vessels in humans (Liebeskind, 2003), which may increase vulnerability to alterations in CBF delivery. Additionally, basal ganglia nuclei, which mediate information processing speed (Cummings, 1998), are some of the most susceptible brain regions to ischemic damage in the context of hypoperfusion (Payabvash et al., 2011) due to being located in the internal watershed region of the brain (Pugh and Lipsitz, 2002). Future research incorporating multi-modal neuroimaging techniques should evaluate regional vulnerabilities to hemodynamic fluctuations and metabolic demands that may result in subsequent structural damage.

While we posit a direct association between cardiac function and brain health to explain the current results, another possible explanation is that findings reflect a top-down phenomenon where early brain changes associated with evolving neuropathology may be responsible for subtle changes in cardiac function. AD pathology develops up to 20 years before clinical symptoms manifest (Bateman et al., 2012) and corresponding chemical changes could affect autonomic nervous system functions (Engelhardt and Laks, 2008). Alternatively, the current findings could be the result of an epiphenomenon or unknown variables that drive changes in both cardiac function and cognition. Additional research is needed to better understand the underlying mechanism(s) accounting for these findings.

Cognitive diagnosis did not modify the association between baseline cardiac output and longitudinal neuropsychological performances. These longitudinal null results align with previous cross-sectional null results (Bown et al., 2019) and suggest that reduced cardiac output has a comparable effect on longitudinal cognitive trajectory regardless of cognitive status. The pathology underlying clinical symptoms in MCI may differ from the processes underlying the association between cardiac output and cognitive decline (Ye et al., 2015). Results presented here may have differed had we assessed our MCI participants years ago prior to clinical symptom onset or if we followed the cohort for a longer period of time (Jefferson et al., 2015).

We observed a subtle interaction between baseline cardiac output and *APOE-*ε4 carrier status on longitudinal information processing speed and episodic memory performances. Interestingly, episodic memory performance localizes to the medial temporal lobes (Tulving and Markowitsch, 1998) while information processing abilities rely on frontal-subcortical circuits (Cummings, 1998). As described above, both the basal ganglia and medial temporal lobes are regions that are susceptible to alterations in blood flow delivery. Therefore, *APOE*-ε4, a moderator of cerebrovascular damage (Halliday et al., 2016), may further increase the susceptibility of the temporal lobes and basal ganglia to damage associated with reduced cardiac output but caution must be taken with such interpretations of the present results. The two significant interactions do not survive correction for false discovery and are attenuated when excluding participants with prevalent CVD and atrial fibrillation, suggesting results may be spurious or driven by less healthy individuals. The mostly null interactions with *APOE-*ε4 carrier status in a longitudinal study of older adults could highlight a phenomenon where *APOE*-ε4 exerts its effects earlier in the aging process and becomes less relevant later in life (Blacker et al., 1997). This age-effect phenomenon is corroborated by recent cross-sectional results of an interaction between cardiac output and *APOE*-ε4 status on cognition for which effects were much more robust (Bown et al., 2019). Additional research is warranted to better understand the intersection of AD genetic risk and vascular health on risk for cognitive decline.

Our study has several strengths, including a longitudinal study design, a community-based cohort free of clinical dementia, stroke, and heart failure at baseline, a comprehensive neuropsychological protocol capturing a diverse range of cognitive outcomes across domains, a reliable imaging technique that reflects the clinical standard for quantifying cardiac output, and a core laboratory for processing all measurements by technicians blinded to clinical information. Additionally, our findings were adjusted for established vascular risk factors and remained consistent in sensitivity analyses excluding participants with prevalent CVD and atrial fibrillation. Despite these strengths, we must note a few limitations. First, our cohort is not reflective of the general population because participants were on average predominantly white, college-educated, older, and relatively cardiac healthy. In fact, participants who withdrew were on average clinically less healthy than participants who were retained over the follow-up, which likely biases our results to the null hypothesis. Generalizability of results to other races, ethnicities, ages, or adults with poorer health is unknown, though it is plausible that in a cohort with more vascular risk factors or cardiac dysfunction, associations might be stronger. Finally, multiple comparisons were used, increasing the likelihood of false-positive findings. We attempted to mitigate this limitation by employing the Benjamini-Hochberg procedure to control for the FDR, and main effect models for language, information processing, and episodic memory continued to meet the significance threshold (*p* < 0.03).

In summary, among community-dwelling older adults, subclinical reductions in systemic blood flow, as assessed by cardiac output, relate to worse longitudinal cognition, particularly in information processing speed, language, visuospatial skills, and episodic memory. Associations were statistically independent of concurrent vascular risk factors captured by FSRP, CVD, or atrial fibrillation and present among a cohort of participants free of clinical stroke, dementia, and heart failure at study entry. Results from the present study argue that vascular measures, such as cardiac output, have important implications in the maintenance of cognitive health with age. Further investigation into the underlying connection between cardiac output and abnormal brain aging may reveal molecular pathways for prevention or therapeutic purposes. Therapies aimed at improving cardiovascular integrity, including lifestyle interventions, should be investigated in more detail as one way to mitigate cognitive decline.

## Data Availability Statement

Due to participant consent restrictions in data sharing, a subset of data is available for purposes of reproducing results or replicating procedures. Data, analytic methods, and study materials can be obtained by contacting the corresponding author.

## Ethics Statement

The studies involving human participants were reviewed and approved by the Vanderbilt University Institutional Review Board. The patients/participants provided their written informed consent to participate in this study.

## Author Contributions

CB and RD: lumped in with the other grouping that contributed analysis and interpretation of data as well as manuscript composition. OK, DL, FC, EM, KO, DG, and LM: analysis and interpretation of data and manuscript composition. KP: acquisition of data, analysis and interpretation of data, study supervision and manuscript composition. TH and KG: study concept and design, acquisition of data, analysis and interpretation of data, study supervision and manuscript composition. AJ: secured funding, study concept and design, acquisition of data, analysis and interpretation of data, study supervision and manuscript composition. All authors contributed to the article and approved the submitted version.

## Conflict of Interest

The authors declare that the research was conducted in the absence of any commercial or financial relationships that could be construed as a potential conflict of interest.

## Footnotes

1 www.r-project.org
