# Supplementary figures and images for: Lower Cardiac Output Relates to Longitudinal Cognitive Decline in Aging Adults
Source: Front Psychol. 2020 Nov 9;11:569355. doi: 10.3389/fpsyg.2020.569355 (PMC7680861; doi:10.3389/fpsyg.2020.569355)

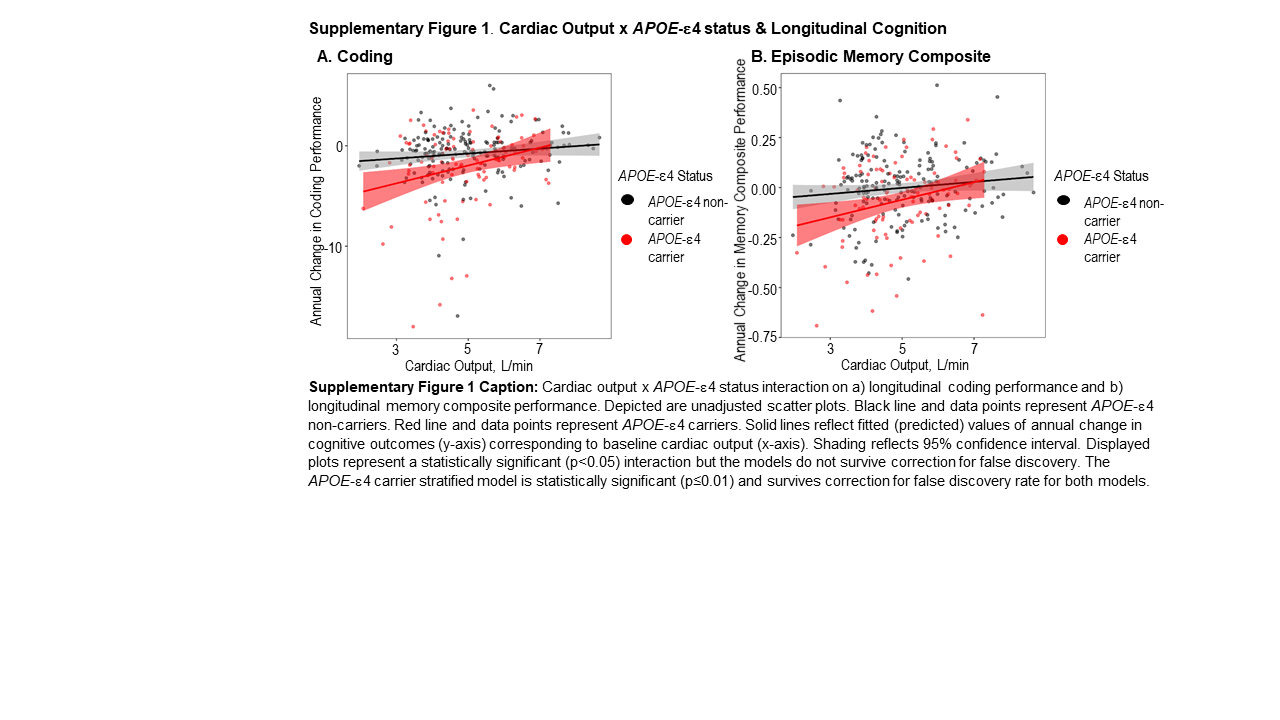

Supplement: Supplementary file 1 [file Image_1.TIF]
