# Supplementary material for: Lower Cardiac Output Relates to Longitudinal Cognitive Decline in Aging Adults
Source: Front Psychol. 2020 Nov 9;11:569355. doi: 10.3389/fpsyg.2020.569355 (PMC7680861; doi:10.3389/fpsyg.2020.569355)
Supplement: Supplementary file 2 [file Table_1.DOCX]

**Supplementary Tables:**

| **Supplementary Table 1. Cardiac Output x Cognitive Diagnosis Interaction and Stratified Models** | | | | | | |
| --- | --- | --- | --- | --- | --- | --- |
|  | **β** | | | **95% Confidence Interval** | | ***p*-value** |
| **Cardiac Output x Cognitive Diagnosis Interaction** |  | | |  | |  |
| Boston Naming Test, 30-Item | 0.07 | | | -0.10, 0.24 | | 0.42 |
| Animal Naming | -0.13 | | | -0.36, 0.10 | | 0.26 |
| WAIS-IV Coding | -0.02 | | | -0.47, 0.42 | | 0.91 |
| DKEFS Number Sequencing^†^ | -0.60 | | | -1.8, 0.56 | | 0.31 |
| Executive Function Composite | 0.006 | | | -0.03, 0.04 | | 0.72 |
| Hooper Visual Organization Test | 0.03 | | | -0.14, 0.20 | | 0.73 |
| Episodic Memory Composite | 0.006 | | | -0.02, 0.04 | | 0.70 |
| **Stratified by NC Participants** | | | | | | |
| Boston Naming Test, 30-Item | | 0.07 | 0.02, 0.12 | | **0.003*** | |
| Animal Naming | | 0.09 | -0.03, 0.22 | | 0.15 | |
| WAIS-IV Coding | | 0.26 | 0.04, 0.48 | | **0.02*** | |
| DKEFS Number Sequencing^†^ | | 0.009 | -0.34, 0.35 | | 0.96 | |
| Executive Function Composite | | 0.003 | -0.008, 0.02 | | 0.56 | |
| Hooper Visual Organization Test | | 0.06 | 0.01, 0.11 | | **0.02*** | |
| Episodic Memory Composite | | 0.02 | -0.001, 0.04 | | 0.07 | |
| **Stratified by MCI Participants** | | | | | | |
| Boston Naming Test, 30-Item | | 0.13 | -0.07, 0.34 | | 0.20 | |
| Animal Naming | | 0.009 | -0.21, 0.22 | | 0.93 | |
| WAIS-IV Coding | | 0.29 | -0.17, 0.74 | | 0.22 | |
| DKEFS Number Sequencing^†^ | | -0.50 | -2.2, 1.2 | | 0.55 | |
| Executive Function Composite | | 0.01 | -0.03, 0.05 | | 0.62 | |
| Hooper Visual Organization Test | | 0.10 | -0.10, 0.30 | | 0.33 | |
| Episodic Memory Composite | | 0.03 | 0.004, 0.05 | | **0.02** | |
| **Note.** Neuropsychological performance values represent the difference between last follow-up visit and baseline visit performances. Data presented as interaction term (cardiac output x time to follow-up). Analyses performed on n=306 participants. Models were adjusted for age, sex, race/ethnicity, education, body surface area, Framingham Stroke Risk Profile minus age, and *APOE-*ε4 status. Bolded values represent significant findings. DKEFS, Delis-Kaplan Executive Function System; MCI, mild cognitive impairment; NC, normal cognition; WAIS-IV, Wechsler Adult Intelligence Scale, 4th edition. All neuropsychological performance values are total correct excluding timed tasks measured in seconds (s). *Models that meet the significance threshold after applying the Benjamini-Hochberg procedure. ^†^Higher values reflect worse performance. | | | | | | |
